# Effects of Host-Specific Multi-Lactic Acid Bacterial Probiotics on Performance, Carcass Traits, Meat Quality, and Gut Microbiome in Fattening Pigs

**DOI:** 10.3390/vetsci13040322

**Published:** 2026-03-26

**Authors:** Katatikarn Sahatsanon, Kamon Chaweewan, Korawan Sringarm, Chaiwat Arjin, Patipan Hnokaew, Apinya Satsook, Premsuda Saman, Hyun-Wook Kim, Pattraphorn Patthararangsarith, Pasin Busayakanit, Kazeem Dauda Adeyemi, Panneepa Sivapirunthep, Chanporn Chaosap

**Affiliations:** 1 Doctoral Program in Innovative Tropical Agriculture, Department of Agricultural Education, School of Industrial Education and Technology, King Mongkut’s Institute of Technology Ladkrabang, Bangkok 10520, Thailand; 65036095@kmitl.ac.th; 2 Bureau of Animal Husbandry and Genetic Improvement, Department of Livestock Development, Pathum Thani 1200, Thailand; krawan2001@gmail.com; 3 Department of Animal and Aquatic Sciences, Faculty of Agriculture, Chiang Mai University, Chiang Mai 50200, Thailand; korawan.s@cmu.ac.th (K.S.); chaiwat.arjin@cmu.ac.th (C.A.); patipan.hnokaew@cmu.ac.th (P.H.); 4 Office of Research Administration, Chiang Mai University, Chiang Mai 50200, Thailand; apinya.satsook@cmu.ac.th; 5 Biodiversity Research Centre, Thailand Institute of Scientific and Technological Research, Pathum Thani 12120, Thailand; premsuda@tistr.or.th; 6 Division of Animal Bioscience & Integrated Biotechnology, Gyeongsang National University, Jinju 52828, Republic of Korea; hwkim@gnu.ac.kr; 7 Department of GreenBio Science, Gyeongsang National University, Jinju 52828, Republic of Korea; 8 Department of Agricultural Education, School of Industrial Education and Technology, King Mongkut’s Institute of Technology Ladkrabang, Bangkok 10520, Thailand; pattraphorn.pa@kmitl.ac.th (P.P.); panneepa.si@kmitl.ac.th (P.S.); 9 Department of Animal Production Technology and Fisheries, Faculty of Agricultural Technology, King Mongkut’s Institute of Technology Ladkrabang, Bangkok 10520, Thailand; 67046026@kmitl.ac.th; 10 Department of Animal Production, Faculty of Agriculture, University of Ilorin, Ilorin PMB 1515, Kwara, Nigeria; adeyemi.kd@unilorin.edu.ng

**Keywords:** swine, intestinal health, ribonucleotide, fatty acids, free amino acids

## Abstract

This study evaluated the effects of a host-specific multi-lactic acid bacterial (MLAB) probiotic and sex on performance, carcass traits, meat quality, and gut microbiota in fattening pigs. Thirty-two crossbred pigs (10 ± 0.80 weeks; 23.43 ± 0.17 kg) were assigned to a 2 × 2 factorial design with diet (control or MLAB probiotics) and sex (barrow or female). The MLAB supplement consisted of seven lactic acid bacterial strains mixed in equal proportions (≈14.3% each)—*Lactobacillus brevis*, *Lactobacillus reuteri*, *Weissella cibaria*, *Lactobacillus paraplantarum*, *Lactococcus lactis*, *Lactobacillus pentosus*, and *Pediococcus pentosaceus*—administered at 1 × 10^9^ CFU/kg feed for 12 weeks. MLAB probiotic supplementation reduced bone proportion while increasing skin and fat content (*p* < 0.05), with a treatment × sex interaction for loin eye area (*p* < 0.05). Meat quality improved in the MLAB group, showing higher ultimate pH and lower cooking loss (*p* < 0.05), indicating improved water-holding capacity. Female pigs exhibited higher early postmortem pH and protein content (*p* < 0.05). Microbiome analysis revealed increased abundances of *Oxalobacteraceae* and *Paludibacteraceae* and reduced *Clostridium sensu stricto* 6 (*p* < 0.05). These results suggest that host-adapted probiotics may support gut microbial balance and improve certain pork quality traits.

## Simple Summary

Most commercial probiotics used in pig production contain bacterial strains that are not originally isolated from pigs. In contrast, host-specific probiotics are obtained directly from the pig gastrointestinal tract, which may allow them to survive better and function more effectively in the pig gut. This study evaluated locally isolated pig-derived probiotic strains as an alternative to conventional commercial probiotic products. The results showed that these host-adapted probiotics did not significantly change growth performance, but they improved pork quality and supported intestinal health by increasing beneficial microorganisms and reducing harmful bacteria. These findings highlight the potential of using probiotics that are naturally adapted to the host species. For producers, this approach may help improve meat quality and maintain gut health while reducing reliance on antibiotics. For consumers, it may contribute to safer and more sustainable pork production.

## 1. Introduction

In modern livestock production, antibiotics have historically been incorporated into animal feeds to prevent disease, modulate intestinal microbiota, and enhance growth performance [1]. However, their excessive use has contributed to the emergence of antibiotic resistance, posing risks to both animal and human health through the consumption of animal-derived foods [2]. As a result, restrictions on antibiotic use in many countries have accelerated the search for effective nutritional strategies that can support animal health and productivity while reducing reliance on antimicrobials [1,2].

Many alternative feed additives to antibiotics have been investigated in swine production, including essential oils, organic acids, phytogenic products, probiotics, and prebiotics [3]. These additives have been reported to improve animal health, growth performance, and intestinal function in pigs [3,4]. Among these alternatives, probiotics have gained increasing attention due to their ability to inhibit pathogenic bacteria in the gastrointestinal tract through the production of antimicrobial substances such as bacteriocins, organic acids, and short-chain fatty acids (SCFAs) [4,5,6].

From a biological perspective, probiotics directly interact with the gut ecosystem and may contribute to improved nutrient utilization and intestinal barrier function. Probiotics are defined as live microorganisms that, when administered in adequate amounts, confer health benefits to the host [2]. Common probiotic candidates, including strains of *Lactobacillus* and *Bifidobacterium*, are frequently derived from the gastrointestinal microbiota of humans and animals [2]. These beneficial microbes may improve gut health by suppressing pathogenic bacteria such as *Escherichia coli* and *Salmonella* through mechanisms including competitive exclusion, reduction of intestinal pH, enhancement of mucosal barrier integrity, and the production of antimicrobial metabolites such as lactic acid and bacteriocins [2]. Moreover, probiotics can directly colonize the gastrointestinal tract and modulate the gut microbial balance, thereby enhancing immune responses and improving intestinal function [6]. Unlike other feed additives such as essential oils, organic acids, and phytogenic products, probiotics can interact with the host microbiota and provide sustained biological effects within the gastrointestinal ecosystem, which may ultimately contribute to improved growth performance in pigs [6,7]. Accordingly, several genera have been widely investigated as probiotics in pigs, including *Bacillus*, *Lactobacillus*, *Bifidobacterium*, *Enterococcus*, *Pediococcus*, and *Streptococcus* [5].

Probiotic supplementation has been associated with multiple benefits across different stages of pig production. In piglets, probiotics may improve growth performance, reduce post-weaning diarrhea, enhance immune function, and promote a balanced gut microbial ecosystem [8]. In fattening pigs, probiotics have also been linked to improvements in carcass traits and pork quality, including favorable postmortem pH, increased cooking yield, reduced drip loss, and enhanced intestinal morphology, such as increased villus height (VH) [9,10,11]. Nevertheless, probiotic efficacy remains highly variable, largely depending on strain characteristics, formulation, and host–microbe interactions [12]. Additionally, pre-slaughter stress such as loading, transportation, lairage, and handling can influence animal welfare, carcass traits and meat quality [13].

Importantly, probiotic functionality is strongly influenced by the ecological origin and host adaptability of the strains. Host-specific probiotics, defined as microorganisms isolated from the same animal species in which they are applied, may exhibit enhanced survivability, colonization potential, and functional compatibility within the gastrointestinal tract compared with non-host-derived commercial strains [14]. Likewise, locally isolated probiotics, obtained from animals raised under regional production conditions, may be better suited to local feeding systems, environmental factors, and microbial ecosystems, thereby improving safety and effectiveness in practical swine production settings. These considerations highlight the importance of identifying probiotic candidates that are not only biologically active but also host-adapted and regionally relevant.

In addition, multi-strain probiotic formulations have attracted increasing interest due to their greater microbial diversity and potential synergistic effects [15]. Compared with single-strain products, multi-strain probiotics may provide broader functionality by combining complementary mechanisms, leading to improved gut health, suppression of pathogens, and enhanced growth and meat quality outcomes [12,15]. Previous studies have evaluated probiotic supplementation in pigs at different physiological stages and production systems. For example, one study demonstrated that supplementation with multi-strain probiotics containing *Lactobacillus acidophilus* (1 × 10^8^ CFU/kg feed) and *Lactobacillus plantarum* (3 × 10^8^ CFU/kg feed) in a 1:1 ratio resulted in greater improvements in growth performance, reduced diarrhea incidence, and enhanced intestinal health compared with single-strain supplementation (*Lactobacillus plantarum* or *Lactobacillus acidophilus*) in weaned pigs during a 21-day experimental period [16]. In growing–finishing pigs, supplementation with 2 g/kg of a multi-strain probiotic consisting of *Lactobacillus plantarum* CJLP243, *Lactobacillus fermentum* LF21, *Lactobacillus salivarius* E4101, *Leuconostoc paramesenteroides* KJP421, *Bacillus subtilis* CJMPB957, and *Bacillus licheniformis* CJMPB283 at approximately 10^9^–10^11^ CFU per strain from 16 to 21 weeks of age over a 6-week experimental period has been reported to promote growth performance and improve intestinal health [4]. In addition, supplementation with a multi-strain probiotic containing *Lactobacillus plantarum* (≥1 × 10^8^ CFU/mL) and *Saccharomyces cerevisiae* (≥0.2 × 10^8^ CFU/mL) at 200 mL/day per sow during the gestation and lactation periods until piglet weaning has been shown to improve carcass characteristics and meat quality in the offspring at 125 days of age [17]. However, despite extensive research, continued identification of novel host-adapted probiotic strains and formulations remains essential, as probiotic effects are highly strain-dependent and context-specific.

Economically, probiotics are relatively easy to incorporate into feed formulations, can be used continuously without withdrawal periods, and have been widely adopted in commercial pig production as a sustainable alternative to antibiotics as a growth promoter.

Despite the increasing use of probiotics in swine nutrition, their efficacy remains inconsistent and highly strain dependent. Although the effects of probiotics in pigs have been widely studied, most research has focused on commercial or single-strain probiotics, and limited information is available regarding host-specific multi-strain probiotic formulations in finishing pigs. Furthermore, it remains unclear whether such formulations can effectively modulate gut microbial communities and translate these changes into improvements in growth performance, carcass traits, and meat quality, particularly considering potential sex-related physiological differences in fattening pigs. To address this gap, seven lactic acid bacterial strains were isolated from healthy Thai pigs based on the work of Saman et al. [18] and supported by the Thailand Institute of Scientific and Technological Research (TISTR). These strains were selected for their probiotic potential, gastrointestinal adaptability, safety, tolerance to gastrointestinal conditions, and adhesion capacity, indicating their suitability as host-specific probiotic candidates. Therefore, the present study evaluated the effects of a host-specific, locally isolated multi-lactic acid bacterial (MLAB) probiotic supplement and sex on growth performance, carcass composition, meat quality, and gut microbiome profiles in fattening pigs. We hypothesized that supplementation with host-specific MLAB probiotics would beneficially modulate the gut microbiome and consequently improve growth performance, intestinal health, carcass composition, and meat quality, with potential differences in response between barrow and female pigs.

## 2. Materials and Methods

### 2.1. MLAB Probiotic Preparation

The freeze-dried probiotic supplement obtained from TISTR contained equal proportions of seven lactic acid bacterial strains: *Lactobacillus brevis* (LM6-9), *Lactobacillus reuteri* (LK33), *Weissella cibaria* (LL5-3), *Lactobacillus paraplantarum* (LM9-3), *Lactococcus lactis* (LL4-5), *Lactobacillus pentosus* (LM3-6), and *Pediococcus pentosaceus* (LL10-5). Each strain accounted for approximately 14.3% of the total bacterial population in the mixture. The supplement was incorporated into the diet of the MLAB group at a concentration of 1 × 10^9^ CFU/kg of feed [17].

The probiotic product was stored according to the TISTR’s recommendations in airtight containers under refrigerated conditions (approximately 4 °C) and protected from moisture and direct light to maintain bacterial viability. The product was incorporated into the diet shortly before feed preparation to minimize viability loss during storage.

### 2.2. Animals, Feeding, and Management

Thirty-two Duroc × (Landrace × Large White) crossbred pigs, aged 10 ± 0.8 weeks with a mean body weight of 23.43 ± 0.17 kg, were used in this study. The sample size was selected based on previous studies evaluating probiotic supplementation in growing pigs [19,20], in which similar numbers of animals per treatment were sufficient to detect effects on gut health and meat quality traits. Pigs were allocated to treatments in a 2 × 2 factorial design, with treatment and sex as the main effects. Prior to allocation, pigs were individually weighed and stratified by body weight and sex to ensure comparable initial conditions between groups. Animals were then randomly assigned to one of two dietary treatments, with 16 pigs per treatment (8 barrows and 8 gilts). The control group received a basal diet, whereas the MLAB group was fed the same basal diet supplemented with a multi-strain probiotic at 1 × 10^9^ CFU/kg of feed. The feeding trial was conducted for 12 weeks. Pigs were housed individually in 1.2 × 1.5 m pens within an evaporative cooling facility. The diet compositions are presented in Table 1. The feeding program consisted of three phases: an initial diet (0–3 weeks) containing 18% crude protein, a grower diet (3–6 weeks) containing 16% crude protein, and a finisher diet (6–12 weeks) containing 14% crude protein.

### 2.3. Growth Performance

Pigs and feed intake were measured on day 0 and at weeks 3, 6, and 12 of the experimental periods to calculate average daily gain (ADG), average daily feed intake (ADFI), and feed and feed conversion ratio (FCR). Feed intake was measured individually for each pig as the difference between feed offered and feed refused, with refusals collected and weighed daily.

### 2.4. Carcass Composition

Pigs were fasted for 12 h, including lairage, before being electrically stunned (220 V for 3–5 s) and slaughtered according to standard procedures: sticking, bleeding, scalding, dehairing, evisceration, and splitting. Hot carcass weight was recorded, and the pH of the right *Longissimus thoracis* (LT) muscle was measured 45 min postmortem (pH_45_). After chilling at 0–4 °C for 18 h, cold carcass weight, carcass length, backfat thickness, and loin eye area (LEA) were measured. Carcass composition was assessed using the Thai-style cutting method [21].

### 2.5. Meat Characteristics

#### 2.5.1. Meat Sample Collection

The right LT muscle of each chilled carcass was measured for ultimate pH (pH_u_) before being cut into four 3 cm thick slices. The first slice was analyzed for physical and chemical properties, including color, water-holding capacity, sarcomere length, and fiber diameter. The second slice was stored at −80 °C for fatty acid, amino acid, and ribonucleotide analyses. The third and fourth slices were then prepared to evaluate the thawing loss, cooking loss, and shear force at different postmortem aging times. For the 1-day aging treatment, the third slices were vacuum-packaged immediately after the initial 24 h chilling period and stored at −20 °C until subsequent meat quality analyses. For the 5-day aging treatment, the fourth slices were vacuum-packaged and stored at 1 °C for an additional 4 days, after which it was transferred to −20 °C until analysis.

#### 2.5.2. pH and Color Measurement

The pH and color were measured following the method described by Chaosap et al. [22]. pH was measured using a calibrated spear-tip pH meter (SG2-ELK SevenGo™, Mettler Toledo, Shanghai, China) with pH 4.01 and 7.01 buffer standards. After a 30 min blooming period, color parameters (CIE L*, a*, b*) were measured in triplicate using a MiniScan EZ spectrophotometer (MiniScan EZ 45/0 LAV, Hunter Associates Laboratory Inc., Reston, VA, USA; D65 illuminant; 8 mm aperture).

#### 2.5.3. Sarcomere Length and Muscle Fiber Diameter Measurement

Sarcomere length and muscle fiber diameter were determined following the procedure of Chaosap et al. [23]. Sarcomere length was measured using a helium–neon laser, whereas muscle fiber diameter was assessed from samples fixed in 4% formalin for 48 h, homogenized in 0.9% NaCl, and examined under 4× magnification using an Olympus CX23 microscope equipped with a Dino-Eye camera system and DinoCapture 2.0 software (v1.5.16C; AnMo Electronics, Taiwan, China).

#### 2.5.4. Water Loss and Shear Force Measurement

Water-holding capacity (WHC) was measured using the press method of Reuter [24] by compressing 0.3 g of meat between filter papers (Whatman no. 1, Shanghai, China) and calculating the meat-to-water area ratio. LT samples, thawed at 4 °C for 24 h, were weighed to determine thawing loss and then cooked at 80 °C until reaching a core temperature of 70 °C to evaluate cooking loss. Shear force was measured on cooked meat strips (1.25 × 3 × 1.25 cm) using a Warner–Bratzler blade attached to a Shimadzu EZ-SX texture analyzer (Shimadzu, Kyoto, Japan).

#### 2.5.5. Proximate Analysis

LT muscle was analyzed for moisture, protein, fat, and ash following AOAC protocols [25]. Moisture content was determined by oven-drying at 105 °C for 24 h; protein content by the Kjeldahl method; fat content by Soxhlet extraction; and ash content by combustion in a muffle furnace at 550 °C for 12 h.

#### 2.5.6. Ribonucleotide Analysis

Ribonucleotide content was determined following the procedure of Chaosap et al. [26]. A 1 g sample was homogenized with 6 mL of 0.6 M perchloric acid, incubated on ice, and centrifuged. The supernatant was filtered, neutralized to pH 7–8, diluted to 25 mL with HPLC-grade water, and centrifuged again before analysis by HPLC (Chromaster, Hitachi, Tokyo, Japan) using a TSKgel Amide-80 column (Tosoh, Tokyo, Japan). The mobile phase consisted of acetonitrile and potassium dihydrogen phosphate (70:30, *v*/*v*), and quantification was performed using external standards. A 1 g sample was homogenized with 6 mL of 0.6 M perchloric acid, incubated on ice, and centrifuged. The supernatant was filtered, neutralized to pH 7–8, diluted to 25 mL with HPLC-grade water, and centrifuged again before analysis by HPLC (Chromaster, Hitachi, Japan) using a TSKgel Amide-80 column (Tosoh, Japan). The mobile phase consisted of acetonitrile and potassium dihydrogen phosphate (70:30, *v*/*v*). Quantification was carried out using external standards, including hypoxanthine, inosine, inosine-5′-monophosphate (IMP), and guanosine-5′-monophosphate (GMP).

#### 2.5.7. Fatty Acid Analysis

Lipids were extracted using the Soxhlet method according to AOAC [25] and analyzed by gas chromatography with flame ionization detection (GC-FID) on a Shimadzu GC-2030 system (Kyoto, Japan). Separation was performed on a fused silica capillary column (RT-2560, Restek, Bellefonte, PA, USA) with helium as the carrier gas. The injector temperature was initially set at 100 °C for 4 min, then increased at 3 °C/min to 240 °C, followed by a 20 min hold. Fatty acid methyl ester standards were used for peak identification based on retention times.

#### 2.5.8. Amino Acid Analysis

Free and total amino acids were analyzed according to ISO [27] and AOAC [25] methods. For free amino acids, 1 g of LT muscle was homogenized in 25 mL of 70% ethanol, centrifuged, and the supernatant was evaporated. The resulting residue was reconstituted in borate buffer for derivatization. Total amino acids were determined by acid hydrolysis (6 M HCl, 120 °C, 24 h), followed by filtration and reconstitution in borate buffer. Both free and total amino acid extracts were derivatized with Fmoc, filtered, and analyzed by HPLC (LC-20A, Shimadzu, Japan) using an Ultra C18 column (Restek, Bellefonte, PA, USA) with fluorescence detection.

### 2.6. Intestinal Morphology

Three segments of the small intestine—the duodenum, jejunum, and ileum—were collected from each pig after slaughter. Samples were fixed in 4% paraformaldehyde solution for 24 h, dehydrated in ethanol, cleared with xylene, and embedded in paraffin. VH and crypt depth (CD) were measured under a compound microscope at 100× magnification, with ten measurements taken per intestinal segment [19].

### 2.7. Microbiome Analysis

#### 2.7.1. DNA Extraction and Amplicon Sequencing

Colonic fecal samples were collected immediately after slaughter and stored at −80 °C. DNA was extracted using the QIAamp Fast DNA Stool Mini Kit (Qiagen, Hilden, Germany). DNA integrity was verified on a 2% agarose gel, and concentrations were measured with a NanoDrop 2000C spectrophotometer (Thermo Scientific, Waltham, MA, USA). The V3–V4 region of the 16S rRNA gene was amplified using primers 341F (5′-CCTAYGGGRBGCASCAG-3′) and 806R (5′-GGACTACNNGGGTATCTAAT-3′) [28]. PCR products were purified with the QIAquick Gel Extraction Kit (Qiagen, Hilden, Germany), quantified using a Qubit 2.0 fluorometer (Thermo Fisher Scientific, Waltham, MA, USA), and sequenced on the Illumina NovaSeq 6000 platform (Illumina Inc., San Diego, CA, USA) to generate 250 bp paired-end reads. The average sequencing depth per sample was 101,760 reads (range: 87,727–121,835 reads). To account for differences in sequencing depth, samples were rarefied to 72,069 reads per sample prior to diversity and taxonomic analyses.

#### 2.7.2. Bioinformatic Analysis

Raw paired-end FASTQ files were demultiplexed using index sequences, and sequence quality was evaluated with FastQC (v0.12.1). Barcode and adapter trimming were performed with Cutadapt (v4.5). Sequence analysis was carried out in QIIME2 (v2023.9), where denoising and merging were performed using DADA2 [29] to generate amplicon sequence variants (ASVs). Taxonomic classification was assigned using the SILVA 138 database at 99% similarity. ASV counts were normalized to the lowest sequencing depth and used for alpha- and beta-diversity analyses.

### 2.8. Statistics

This experiment was conducted using a 2 × 2 factorial design with two dietary treatments (control and MLAB) and two sexes (barrow and female). The statistical model used for data analysis was as follows:
Yijk = µ + Bi + Aj + BiAj + €ijkµ = the overall mean excluding experimental influencesBi = the effect of i in sexes, when i = barrows and giltsAj = the effect of j in treatments, when j = control and MLAB groupsBiAj = the interaction effect between treatments and sexes€ijk = the random error associated with each observation

Data were analyzed using two-way analysis of variance (ANOVA) to evaluate the main effects of dietary treatment and sex, as well as their interaction. Prior to interpreting the ANOVA results, the assumptions of the model were evaluated. A two-way ANOVA model was first fitted to the data, and residuals from the fitted model were obtained for diagnostic assessment. The normality of residuals was evaluated using the Shapiro–Wilk test and visual inspection of Q–Q plots. Homogeneity of variances among treatment groups was assessed using Levene’s test. Independence of observations was ensured through the experimental design and random allocation of treatments. When a significant interaction between diet and sex was detected (*p* < 0.05), simple effect analyses were performed to evaluate the effect of one factor within each level of the other factor, and the corresponding means were reported accordingly. When no significant interaction was observed (*p* ≥ 0.05), only the main effects of diet and sex were interpreted and presented. For significant main effects, differences among means were separated using Tukey’s multiple comparison test. Differences were considered statistically significant at *p* < 0.05. All statistical analyses were performed using SPSS software (version 29).

Microbial diversity was analyzed in QIIME2. Chao1 and Shannon indices were computed for alpha diversity, with group comparisons conducted using the Mann–Whitney U test in R (v4.3.1). Beta diversity was assessed with Bray–Curtis distances and permutational multivariate analysis of variance (PERMANOVA) and visualized by principal coordinates analysis (PCoA). Taxonomic differences between groups were further evaluated using the Mann–Whitney U test, and *p*-Values were adjusted for multiple comparisons using the Benjamini–Hochberg false discovery rate (FDR) method.

### 2.9. Data Availability

All 16S rRNA sequencing data are available in the NCBI SRA under BioProject ID PRJNA1189043 at https://www.ncbi.nlm.nih.gov/sra (accessed on 21 November 2024).

## 3. Results

### 3.1. Growth Performance

The interaction between dietary treatment and sex had no significant effect on growth performance parameters, including body weight, ADG, ADFI, and FCR (*p* > 0.05; Table 2). A tendency for higher ADG during weeks 0–3 was observed in the MLAB group (*p* = 0.06); however, the difference was not statistically significant. MLAB probiotic supplementation or sex had no significant impact on pig performance (*p* > 0.05).

### 3.2. Carcass Composition

**Figure 1:**
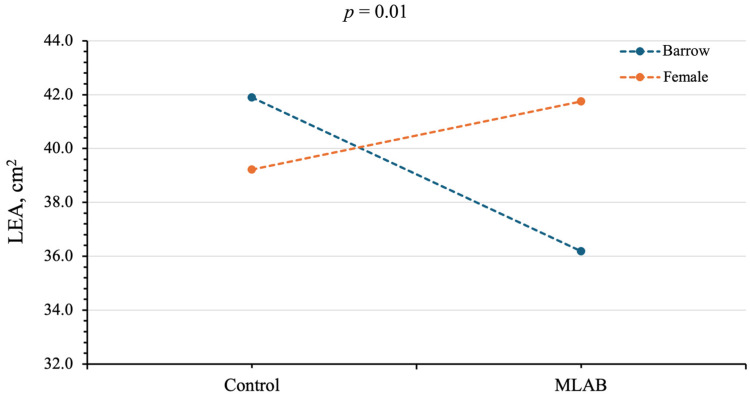
Interaction between treatments (control and multi-lactic acid bacterial probiotics) and sex (barrow and female) on loin eye area (LEA). A *p*-Value < 0.05 was considered statistically significant.

A significant interaction between dietary treatment and sex was observed for LEA (*p* < 0.05; Figure 1). MLAB probiotic supplementation resulted in a significantly lower LEA in barrow pigs (36.18 cm^2^) than in female pigs (41.75 cm^2^), whereas no difference in LEA was observed between barrow and female pigs in the control group (41.90 and 39.22 cm^2^, respectively; Figure 1). The main effect of dietary supplementation did not significantly affect final weight, hot carcass weight, chilled carcass weight, carcass length, backfat thickness, LEA, or lean meat percentage (*p* > 0.05; Table 3), except for bone and skin and fat percentages. Pigs receiving MLAB had a lower bone proportion than those in the control group (18.60% vs. 19.41%), whereas the MLAB group showed a higher proportion of skin and fat compared with the control group (18.61% vs. 17.27%) (*p* < 0.05). Carcass characteristics were not significantly affected by sex (*p* > 0.05; Table 3).

### 3.3. Meat Characteristics

The interaction between dietary treatment and sex was not significant for meat quality variables (*p* > 0.05; Table 4). Therefore, the main effects of each factor are presented as follows. MLAB probiotic supplementation resulted in a significantly higher pH_u_ and lower cooking loss at 1 day postmortem (5.56 and 19.49%, respectively) compared with the control group (5.47 and 21.94%, respectively; *p* < 0.05). In addition, a tendency toward reduced WHC was observed in the MLAB group (0.26 vs. 0.30; *p* = 0.07). Female pigs showed a significantly higher pH_45_ value than barrow pigs (6.24 vs. 6.02, respectively; *p* < 0.05).

### 3.4. Proximate and Ribonucleotide Composition

There was no significant interaction between treatment and sex for proximate composition or ribonucleotides (*p* > 0.05; Table 5). Thus, the main effects of each factor were evaluated independently. The sex factor significantly increased the protein content, with higher values observed in female pigs than in barrow pigs (24.10 vs. 22.80%, *p* < 0.05).

### 3.5. Fatty Acid Composition

The interaction between dietary treatment and sex was not significant for fatty acid composition (*p* > 0.05; Table 6); therefore, the main effects of each factor were evaluated. MLAB probiotic supplementation significantly reduced eicosadienoic acid (C20:2) compared with the control group (0.27 vs. 0.40; *p* < 0.05). Sex did not significantly affect the fatty acid composition of the LT muscle (*p* > 0.05; Table 6).

### 3.6. Amino Acid Composition

The interaction between treatment and sex was significant for serine free amino acids (*p* < 0.05; Table 7). Neither treatment nor sex significantly affected the total amino acid composition of the LT muscle (*p* > 0.05; Table 7).

**Figure 2:**
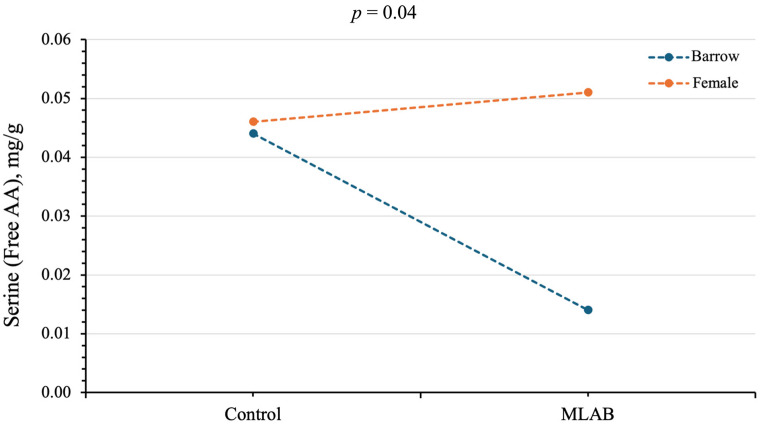
Interaction between treatments (control and multi-lactic acid bacterial probiotics) and sex (barrow and female) on free amino acid (Free AA) serine. A *p*-Value < 0.05 was considered statistically significant.

Significant interaction between treatment and sex was significant for free amino acid serine (*p* < 0.05; Figure 2). MLAB probiotic supplementation decreased serine content in barrow pigs (0.01 mg/g) but increased it in female pigs (0.05 mg/g; Figure 2). In contrast, serine content did not differ between barrow and female pigs in the control group (0.044 and 0.046 mg/g, respectively; Figure 2). The free amino acid composition and human taste classification were not significantly affected by dietary factors (*p* > 0.05; Table 8). However, sex influenced some free amino acids, including glutamic acid, proline, and umami taste (Table 8). Barrow pigs showed significantly lower levels of glutamic acid, but higher levels of proline compared with female pigs (0.10 vs. 0.20 and 0.08 vs. 0.05, respectively; *p* < 0.05). Umami-related amino acids were significantly lower in barrow pigs compared to female pigs (0.10 vs. 0.20; *p* < 0.05).

### 3.7. Intestinal Morphology

The interaction between two experimental factors did not significantly affect intestinal morphology (*p* > 0.05; Table 9). Thus, the main effect of dietary and sex treatments was evaluated independently. MLAB probiotic supplementation did not significantly affect intestinal morphology measures (VH, CD, VH:CD ratios; *p* > 0.05; Table 9). Barrow pigs showed a significantly higher VH:CD ratio in the duodenum and jejunum (1.91 and 2.31 µm, respectively) than female pigs (1.48 and 1.86 µm, respectively; *p* < 0.05), whereas CD in the ileum was significantly lower in barrow pigs (162.02 µm) than in female pigs (202.24 µm; *p* < 0.05).

### 3.8. Gut Microbiome

**Figure 3:**
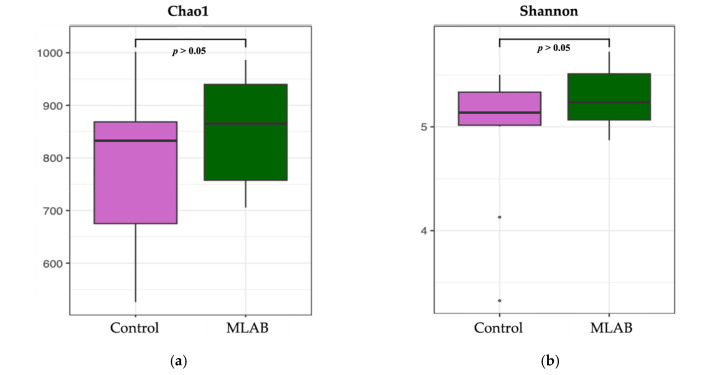

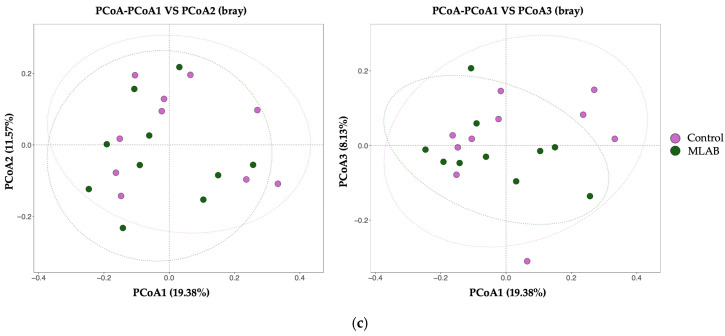
Analysis of the gut microbiota. Diversity and richness of microorganisms in piglet feces between the control (*n* = 10) and multi-lactic acid bacterial probiotics (*n* = 10) groups, assessed by (**a**) Chao1 and (**b**) Shannon indices. (**c**) Beta diversity of fecal microbiota based on Bray–Curtis distances and visualized by principal coordinates analysis (PCoA). Chao1 and Shannon indices were compared using the Mann–Whitney U test, and beta diversity was analyzed using permutational multivariate analysis of variance. A *p*-Value < 0.05 was considered statistically significant.

Microbial richness and diversity, evaluated using Chao1 and Shannon indices, did not differ significantly between treatment groups (*p* > 0.05, Figure 3a,b). In addition, PCoA showed no distinct separation between groups, suggesting comparable microbial community composition (Figure 3c).

**Figure 4:**
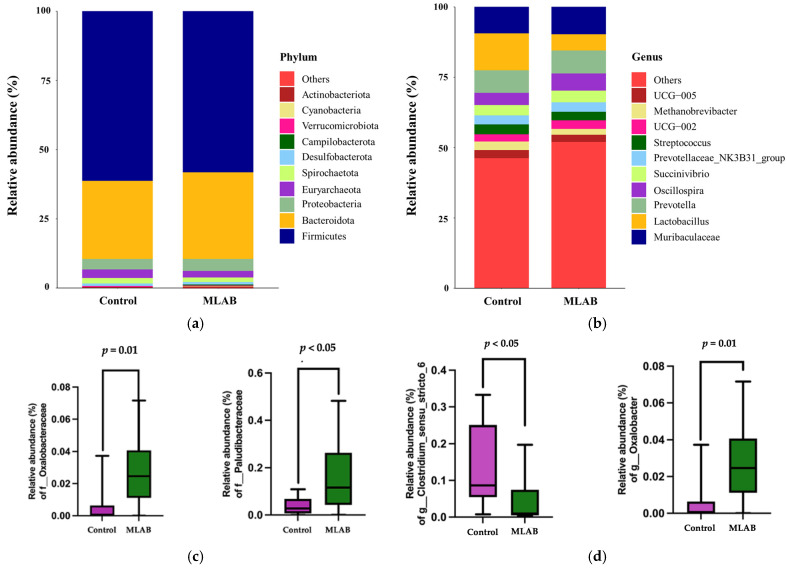
Relative abundance of microbial taxa. Histograms showing differences in the relative abundance of microbial taxa in piglet feces at the (**a**) phylum and (**b**) genus levels between the control (*n* = 10) and multi-lactic acid bacterial probiotics (*n* = 10) groups. Statistical differences in the fecal microbial community were assessed using the Mann–Whitney U test at the (**c**) family and (**d**) genus levels. A *p*-Value < 0.05 was considered statistically significant.

*Firmicutes* (61.36% vs. 58.28%) and *Bacteroidota* (28.13% vs. 31.33%) were the dominant microbial phyla, with only minor differences in their relative abundances between control and MLAB groups, respectively (Figure 4a). Other phyla, including *Campylobacterota*, *Verrucomicrobiota*, *Cyanobacteria*, and *Actinobacteriota*, were detected at levels below 1%. The top ten microbial taxa at both the phylum and genus levels are presented in Supplementary Table S1. *Lactobacillus* was more abundant in the MLAB group (13.24%) than in the control (5.77%), whereas *Oscillospira* was present at lower levels (4.37% vs. 6.21%) (Figure 4b). The Mann–Whitney U test indicated significantly higher relative abundances of the families *Oxalobacteraceae* (0.03% vs. 0.01%, *p* = 0.01) and *Paludibacteraceae* (0.17% vs. 0.04%, *p* < 0.05) in the MLAB group compared with the control group (Figure 4c). Among 5 differentially abundant genera (Supplementary Table S2), *Clostridium sensu stricto 6* decreased (0.038% vs. 0.120%, *p* < 0.05), whereas *Oxalobacter* increased (0.028% vs. 0.005%, *p* = 0.01) in pigs fed MLAB compared with the control group (Figure 4d).

## 4. Discussion

This study aimed to evaluate the effects of a host-specific, locally isolated multi-lactic acid bacterial (MLAB) probiotic supplement and sex on growth performance, carcass composition, meat quality, and gut microbiome profiles in fattening pigs. The results contribute to a better understanding of the potential role of MLAB probiotics and sex differences in shaping productive performance and gut microbial ecology in fattening pigs.

Growth performance was unaffected by MLAB probiotic supplementation during both the growing and fattening phases. This is partially consistent with Duan et al. [9], who reported that *Lactococcus lactis* improved pig performance during the growing phase but not during the finishing phase. Previous studies suggest that mixed probiotics can enhance growth by supporting gut health, nutrient absorption, and immune function [4,9,12]. Differences in response may result from variations in gut microbiota, age, diet, or probiotic strains [15]. Moreover, the immature gut microbiome of younger pigs may be more responsive to probiotics, which could explain the reduced effect observed in later stages [9].

The interaction between treatment and sex significantly affected LEA (*p* < 0.05). MLAB probiotic supplementation decreased LEA in barrow pigs but increased LEA in female pigs, while control group barrow and female pigs were not different. Previous studies have reported that gilts generally exhibit a larger LEA than barrows, reflecting their greater lean tissue deposition [30]. Therefore, the observed interaction may indicate that barrow and female pigs respond differently to dietary supplementation in terms of muscle development, which may be associated with sex-related variations in protein deposition and muscle growth. MLAB-supplemented pigs had less bone but more skin and fat than controls (*p* < 0.05). This finding is consistent with Zhu et al. [17], who reported that the effects of probiotics on fat deposition vary with age, initially reducing but later increasing fat accumulation. Schumacher et al. [31] suggested that fat accumulation tends to rise when muscle and bone growth decline, as excess dietary energy is redirected toward fat storage. A possible explanation is that probiotic supplementation may influence microbial fermentation in the gut, potentially altering the production of short-chain fatty acids (SCFAs) such as acetate, propionate, and butyrate [32,33]. Acetate has been suggested to serve as a substrate for lipogenesis and may therefore contribute to fat deposition in pigs [34]. However, because SCFA concentrations were not determined in the present study, this mechanism remains hypothetical and requires further investigation. However, the magnitude of bone differences was relatively small. The bone percentage decreased by only 0.81%, while skin and fat increased by 1.34% in the MLAB group compared with the control group. From a practical perspective, such minor differences are unlikely to substantially influence carcass value or economic returns in commercial pork production, where overall carcass weight, lean yield, and meat quality traits are generally considered more important indicators of profitability.

Postmortem glycolysis lowers muscle pH by converting glycogen into lactic acid, which in turn affects WHC, color, and tenderness [17]. WHC, the ability of meat to retain its intrinsic water, is a crucial determinant of meat quality [35]. Similarly, pHu is an important quality indicator, reflecting the degree of postmortem glycogen depletion and strongly influencing meat attributes such as color, tenderness, water loss, and shelf life [36]. This study found that female pigs had higher pH_45_ compared with barrow pigs (*p* < 0.05). Similarly, Xia et al. [37] reported higher pH_45_ values in female pigs than in males, suggesting that sex may influence glycogen metabolism and the rate of post-mortem glycolysis [38]. Currently, studies examining the influence of sex on specific pork quality traits remain relatively limited. Therefore, further studies are needed to confirm these findings. In addition, MLAB probiotic supplementation increased pHu and reduced cooking loss at one day postmortem (*p* < 0.05), whereas other meat quality traits were unaffected. However, the tendency toward lower WHC appears partly inconsistent with the increased pHu and reduced cooking loss. This discrepancy may be explained by differences in measurement principles: the press method estimates expressible water under mechanical pressure, whereas cooking loss reflects water loss during thermal processing. Because cooking loss is largely influenced by heat-induced protein denaturation, it may respond differently to dietary treatments than water retention measured under pressure. One possible explanation for the higher pHu and lower cooking loss observed in this study is that probiotic supplementation may influence postmortem glycolytic metabolism, although this mechanism was not directly examined. Previous studies have suggested that glycolytic enzymes such as β-enolase (ENO3) and pyruvate kinase (PKM2) regulate lactic acid production and the rate of pH decline during postmortem metabolism [39]. However, because enzyme activity or gene expression was not determined in the present study, this interpretation should be considered speculative. Consistent with this hypothesis, Zhu et al. [17] reported improved pH and cooking yield following supplementation with *Lactobacillus plantarum* and *Saccharomyces cerevisiae*, whereas *Lactobacillus reuteri* did not produce similar effects [10].

Another possible explanation for the improvement in specific meat quality traits, such as pHu and water retention, may be related to reduced physiological stress in probiotic-supplemented pigs. Blood cortisol and creatine kinase (CK) are commonly used indicators of stress in pigs [40]; however, their relationships with pork quality traits are often inconsistent. Probiotic supplementation has been reported to reduce cortisol levels and improve immune function in weaning pigs, thereby supporting better health and growth [41]. Shaw and Thaw [42] reported that serum and muscle cortisol were negatively correlated with drip loss and positively correlated with ultimate pH (*p* < 0.05). In contrast, Koomkrong et al. [43] found that drip loss was primarily influenced by postmortem muscle pH decline, with higher white blood cell and monocyte counts observed in pigs with lower drip loss, suggesting a potential association between immune status and water-holding capacity, whereas cortisol was not significantly related to drip loss. Similar weak or inconsistent relationships between physiological stress markers and pork quality characteristics have been reported under commercial production conditions, indicating that cortisol and CK measured at slaughter may have limited predictive value for technological and sensory meat quality traits [44,45]. Therefore, pork quality outcomes are likely influenced by multiple interacting factors, including muscle metabolism, genetics, immune status, and pre-slaughter handling conditions.

Sarcomere length ranged from 2.50 to 2.53 μm, showing no significant differences between groups and remaining within the normal physiological range [46]. Muscle fibers, which account for 70–90% of skeletal muscle mass, play a major role in pork quality by affecting texture, moisture retention, and sensory properties [10]. Although smaller fiber diameters are generally associated with improved WHC and reduced drip loss, MLAB probiotic supplementation had no effect on this trait. This contrasts with Tian et al. [10], who reported smaller myofibers in pigs supplemented with *Lactobacillus reuteri* 1.

The nutritional composition of meat, including protein, fat, and moisture content, was evaluated using proximate analysis. Consistent with Chang et al. [47], who found no significant changes following *Lactobacillus plantarum* supplementation, our results showed no major differences between groups. However, pigs receiving the MLAB-supplemented diet tended to have slightly lower protein content. In this study, barrow pigs showed lower protein content in the LT muscle than female pigs (*p* < 0.05). Previous studies have indicated that the effect of sex on pork proximate composition is generally small. For example, Lorenzo et al. [48] and Żmijewski and Modzelewska-Kapituła [49] reported that sex did not significantly affect the proximate composition of pork. Alves et al. [50] also reported higher protein content in female pigs than in male pigs, although the difference was not statistically significant. The higher protein content observed in female pigs in the present study may be related to a higher lean-to-fat ratio in females, whereas barrows generally accumulate more fat, which may reduce the relative protein content in the muscle [51].

After slaughter, nucleotide triphosphates such as adenosine triphosphate (ATP) and guanosine triphosphate (GTP) break down into flavor-related compounds like IMP and GMP. IMP enhances umami, hypoxanthine imparts bitterness, and inosine is tasteless [52,53]. Ribonucleotides, particularly adenosine monophosphate (AMP), IMP, and GMP, play a key role in enhancing umami and overall meat flavor perception [53]. However, this study did not detect any significant variation in ribonucleotide content between treatments. Similarly, Chang et al. [47] also found that ribonucleotide content remained unaffected by *Lactobacillus plantarum*.

The fatty acid profiles (SFA, MUFA, and PUFA) were similar between groups and fell within the typical pork fat range of approximately 38% SFA, 50% MUFA, and 12% PUFA [54]. However, the control showed significantly higher levels of eicosadienoic acid (C20:2) and numerically higher levels of eicosatrienoic acid (C20:3n3), which are associated with inflammation [55]. This may reflect the anti-inflammatory effects of probiotics, which can influence fatty acid synthesis and metabolic pathways involved in inflammatory processes [56]. Similarly, Tian et al. [10] found that *Lactobacillus reuteri* 1 supplementation reduced docosahexaenoic acid (C22:6) levels without significantly altering SFA, MUFA, or PUFA content. Grela et al. [57] also reported no significant changes in SFA or MUFA, but observed a decrease in PUFA levels in pigs fed a probiotic mixture.

Pork flavor and nutritional quality are influenced by factors such as genetics, diet, age, and husbandry practices [10,58]. Meat contains essential nutrients, including proteins, carbohydrates, and lipids, with amino acids playing a key role in both muscle synthesis and flavor development [59]. Total amino acids reflect nutritional value, while free amino acids contribute directly to taste. Aspartic acid and glutamic acid enhance umami; threonine, serine, glycine, alanine, and proline impart sweetness; and histidine, threonine, lysine, valine, methionine, isoleucine, and tryptophan contribute bitterness [59]. In this study, the interaction effect between treatment and sex was influential for serine free amino acids (*p* < 0.05). MLAB probiotic supplementation decreased serine in barrow pigs, whereas serine increased in female pigs. Free amino acids contribute directly to meat flavor, and serine is involved in several metabolic processes, including growth, protein metabolism, and amino acid synthesis [60]. Therefore, the higher serine concentration observed in female pigs may be associated with differences in muscle protein metabolism between sexes. Both sex and MLAB probiotic supplementation affected the serine content. Qin et al. [61] reported that the concentrations of free amino acids glutamic acid and proline in the *longissimus* muscle of gilts ranged from 12.11 to 13.63 and 12.80 to 13.19 µg/g, respectively. These findings suggest that the effect of MLAB probiotic supplementation on serine metabolism may differ between sexes. Further studies are needed to clarify the mechanisms by which MLAB influences serine metabolism, particularly in female pigs. In addition, sex effect showed that female pigs had higher levels of glutamic acid and umami-related amino acids, but lower levels of proline than barrow pigs (*p* < 0.05). Glutamic acid is a key contributor to the umami taste and may partly explain the higher concentration of umami-related amino acids observed in female pigs [62]. The dietary effect did not significantly affect either total or free amino acid content. In contrast, Chang et al. [47] reported that *Lactobacillus plantarum* reduced specific free amino acids, including serine, lysine, histidine, and arginine, leading to decreased bitterness, and Tian et al. [10] observed reductions in isoleucine, leucine, methionine, and proline with *Lactobacillus reuteri* 1, without affecting total amino acid levels. These results suggest that sex-related differences in muscle metabolism may contribute to the variation in certain free amino acids.

The small intestinal villi are essential for increasing surface area and enhancing nutrient absorption [63]. Jejunal VH tended to be higher in the MLAB group (*p* = 0.09). Joysowal et al. [19] found that single-strain probiotics like *Lactobacillus acidophilus* or *Pediococcus acidilactici* FT28 improved jejunal morphology by increasing both VH and CD. Similarly, Kwak et al. [4] reported that a multi-strain probiotic mixture increased VH and VH:CD ratio while reducing CD in pigs. These changes may result from enhanced feed intake driven by *Lactobacillus* spp., which stimulate intestinal epithelial development [17]. Improved intestinal structure supports better nutrient absorption and overall gut health, both vital for optimal pig growth and digestion [19,63]. In the present study, sex also influenced intestinal morphology, as female pigs showed a lower VH:CD ratio in the duodenum and jejunum and greater CD compared with barrow pigs. Feed intake is an important factor affecting intestinal structure, including VH, CD, and intestinal length [64]. Previous studies have reported that male pigs generally exhibit higher body weight and feed intake than female pigs [65], which may contribute to improved intestinal structure in males. In addition, an increase in VH, a reduction in CD, and, consequently, a higher VH:CD ratio are generally considered indicators of improved gut health [66].

Gut microorganisms are essential for digestion and nutrient absorption, and microbial diversity is widely regarded as an important indicator of host health [20]. In this study, alpha diversity was assessed using the Chao1 index for species richness and the Shannon index for microbial diversity [63], with no significant differences observed between the MLAB and control groups. Li et al. [20] similarly found that adding *Bacillus subtilis* did not alter these parameters in growing pigs. These findings suggest that probiotics may influence specific microbial populations without altering overall diversity, particularly in animals with an established and resilient gut microbiota.

Gut microorganisms play a crucial role in digestion and nutrient absorption, with microbial diversity widely considered a key indicator of host health [20]. In the present study, alpha diversity was evaluated using the Chao1 index to estimate species richness and the Shannon index to assess overall microbial diversity [63]. No significant differences were observed between the MLAB and control groups. Consistent with these findings, Li et al. [20] reported that supplementation with *Bacillus subtilis* did not alter these diversity metrics in growing pigs. Collectively, these results suggest that probiotics may modulate specific microbial populations without substantially affecting overall gut diversity, particularly in animals with a well-established and resilient microbiota. These results may be consistent with the concept of host-adapted nutritional strategies, in which nutritional interventions tailored to the host can improve gut function in livestock [67]. In this study, the MLAB probiotic strains were isolated from healthy Thai pigs, which may enhance their adaptation to the host gut environment.

This study found higher abundances of *Oxalobacteraceae* and *Paludibacteraceae* in the MLAB group. Ma et al. [68] reported that *Oxalobacteraceae* is associated with bacterial diversity and consists primarily of beneficial bacteria. *Paludibacteraceae*, a family within the phylum *Bacteroidota* [69], is commonly present in the gut of pigs and other animals. Although not as extensively studied as other microbial families, emerging evidence suggests that it plays a role in immune modulation [70]. Specifically, *Paludibacteraceae* may contribute to host immune regulation through its metabolic activity. SCFAs such as acetate and propionate, produced by members of this family, are recognized for their anti-inflammatory properties and for supporting intestinal barrier integrity [71]. These SCFAs interact with host immune cells, modulating cytokine production and helping to maintain immune balance [72]. At the genus level, the MLAB group showed lower levels of *Clostridium sensu stricto* 6 and higher levels of *Oxalobacter*. *Oxalobacter formigenes*, a key member of this genus, is essential for gut health by breaking down oxalate, which can be toxic to animals when present in excess [73,74]. Unlike most gut bacteria, *Oxalobacter* relies solely on oxalate as its energy source [74]. The increased abundance of *Oxalobacter* in the MLAB group suggests enhanced oxalate metabolism. Conversely, the reduced levels of potentially harmful *Clostridium sensu stricto* 6 may reflect improved gut health.

## 5. Conclusions

Host-specific and locally isolated multi-lactic acid bacterial probiotic supplementation did not affect growth performance in fattening pigs, but it improved the quality of certain types of pork, resulting in higher ultimate pH and lower cooking loss. The probiotic supplementation also changed carcass composition and altered the relative abundance of several intestinal bacterial groups, suggesting a potential role in supporting intestinal microbial balance. These findings indicate that host-adapted probiotic formulations may represent a promising nutritional strategy to modulate gut microbial communities and improve specific pork quality characteristics in fattening pigs. However, the interpretation of the relationship between intestinal microbial changes and carcass or meat quality traits should be made cautiously because the study involved a relatively limited number of animals and did not evaluate functional microbial metabolites. Further studies with larger populations and functional analyses are required to better clarify the mechanisms underlying these responses. These findings suggest that host-specific probiotic formulations may provide a practical nutritional approach to improve certain pork quality traits and support intestinal microbial balance in fattening pigs, even in the absence of changes in growth performance.

## Figures and Tables

**Table 1:** Ingredient composition and chemical compositions in experimental diets.

Items	18% CP	16% CP	14% CP
Ingredients (%)			
Broken rice	53.00	51.00	53.00
Rice bran	15.00	22.40	26.10
Soybean meal	22.00	19.50	15.00
Fish meal	5.00	2.00	0.80
Dicalcium phosphate	4.00	4.00	4.00
Salts	0.20	0.50	0.50
L-Lysine	0.10	0.10	0.10
Premix	0.25	0.25	0.25
Mycotoxin binder	0.20	0.20	0.20
Feed additives	0.05	-	-
Antibiotic	0.07	-	-
Vitamin C	0.10	0.05	0.05
Anthelmintic	0.03	-	-
Total	100.00	100.00	100.00
Calculated analysis (%)			
Crude protein	18.31	16.42	14.44
Ether extract	3.19	3.69	3.6
Crude fiber	3.59	3.79	3.48
Total calcium	0.91	0.85	0.62
Total phosphorus	0.71	0.59	0.49
Lysine	1.09	0.89	0.76
Methionine	0.36	0.33	0.28
ME (kcal/kg)	3122.36	3197.84	3236.89

Note: Premix, vitamin A 8,000,000 IU+ vitamin D 1,500,000 IU+ vitamin E 40,000 ppm+ V vitamin K 1500 ppm+ thiamin 1000 ppm+ riboflavin 4000 ppm+ vitamin B12 20 ppb+ pyridoxine 2000 ppm+ niacin 20,000 ppm+ biotin 30 ppm+ folic acid 600 ppm+ Se 250 mg+ I 200 mg+ Fe 60,000 mg+ Mn 25,000 mg+ Zn 60,000 mg+ Cu 15,000 mg (supplied/kg diet); ME, metabolizable energy.

**Table 2:** Effects of multi-lactic acid bacterial (MLAB) probiotic supplementation on the growth performance of fattening pigs.

Trait	Treatment (T)		Sex (S)		SE	*p*-Value		
	Control	MLAB	Barrow	Female		T	S	T × S
Body weight (kg)								
Initial body weight	23.11	23.59	23.43	23.26	0.89	0.70	0.89	0.34
Week 3	36.88	36.06	36.78	36.16	1.53	0.71	0.78	0.34
Week 6	56.33	55.38	55.88	55.83	2.03	0.74	0.99	0.54
Week 12	103.10	101.94	103.06	101.98	2.15	0.72	0.73	0.82
Week 0–3								
BWG (kg)	13.77	12.47	13.34	12.89	1.02	0.38	0.76	0.54
ADG (kg/d)	0.80	0.68	0.75	0.73	0.04	0.06	0.74	0.98
ADFI (kg/d)	1.65	1.57	1.61	1.61	0.03	0.08	0.94	0.98
FCR	2.11	2.49	2.22	2.38	0.20	0.17	0.57	0.54
Week 3–6								
BWG (kg)	19.45	19.31	19.09	19.67	0.83	0.91	0.63	0.79
ADG (kg/d)	0.90	0.88	0.88	0.89	0.03	0.65	0.83	0.98
ADFI (kg/d)	2.17	2.11	2.15	2.13	0.06	0.51	0.84	0.92
FCR	2.44	2.45	2.49	2.40	0.11	0.98	0.59	1.00
Week 6–12								
BWG (kg)	46.78	46.56	47.19	46.15	1.10	0.90	0.53	0.51
ADG (kg/d)	1.13	1.14	1.14	1.13	0.02	0.81	0.72	0.24
ADFI (kg/d)	3.98	4.06	3.96	4.08	0.07	0.40	0.23	0.33
FCR	3.54	3.60	3.50	3.64	0.10	0.67	0.35	0.92
Overall								
BWG (kg)	79.99	78.34	79.63	78.71	1.75	0.53	0.73	0.84
ADG (kg/d)	1.00	0.96	0.99	0.97	0.02	0.27	0.68	0.40
ADFI (kg/d)	2.41	2.40	2.38	2.43	0.03	0.94	0.38	0.32
FCR	2.42	2.50	2.42	2.50	0.04	0.23	0.24	0.86

Note: MLAB, multi-lactic acid bacterial; BWG, body weight gain; ADG, average daily gain; ADFI, average daily feed intake; FCR, feed conversion ratio; SE, standard error. A *p*-Value < 0.05 was considered statistically significant.

**Table 3:** Effects of multi-lactic acid bacterial (MLAB) probiotic supplementation on carcass characteristics of fattening pigs.

Items	Treatment (T)		Sex (S)		SE	*p*-Value		
	Control	MLAB	Barrow	Female		T	S	T × S
Final weight (kg)	112.78	114.19	112.91	114.06	0.85	0.25	0.34	0.43
Hot carcass weight (kg)	85.70	86.06	85.56	86.20	0.92	0.78	0.63	0.90
Hot carcass yield (%)	76.01	75.37	75.80	75.58	0.67	0.50	0.82	0.42
Chilled carcass weight (kg)	83.34	84.06	83.31	84.09	0.90	0.57	0.54	0.99
Chilled carcass yield (%)	73.92	73.62	73.80	73.73	0.68	0.76	0.94	0.51
Carcass length (cm)	100.88	100.25	99.50	101.63	0.89	0.62	0.10	0.49
Backfat thickness (inch)	1.12	1.21	1.19	1.14	0.03	0.06	0.31	0.07
Loin eye area (cm^2^)	40.56	38.97	39.04	40.48	1.10	0.36	0.31	0.01
Lean meat (%)	39.20	38.33	38.67	38.86	0.39	0.13	0.73	0.22
Bone (%)	19.41	18.60	19.24	18.77	0.14	0.01	0.12	0.85
Skin and fat (%) ^1^	17.27	18.61	18.22	17.66	0.24	<0.001	0.11	0.05

Note: MLAB, multi-lactic acid bacterial; SE, standard error; **^1^** % of chilled carcass. A *p*-Value < 0.05 was considered statistically significant.

**Table 4:** Effects of multi-lactic acid bacterial (MLAB) probiotic supplementation on meat quality of fattening pigs.

Items	Treatment (T)		Sex (S)		SE	*p*-Value		
	Control	MLAB	Barrow	Female		T	S	T × S
pH_45_	6.07	6.20	6.02	6.24	0.07	0.20	0.03	0.06
pH_u_	5.47	5.56	5.51	5.52	0.03	0.04	0.71	0.25
Color								
L*	56.32	56.44	56.37	56.39	0.52	0.88	0.98	0.41
a*	4.23	3.63	3.85	4.01	0.27	0.13	0.69	0.63
b*	13.89	13.85	13.89	13.85	0.24	0.91	0.18	0.67
WHC	0.30	0.26	0.27	0.29	0.02	0.07	0.34	0.69
Thawing loss (%)								
1-day postmortem	6.59	5.57	6.51	6.65	0.47	0.13	0.21	0.69
5-day postmortem	4.52	4.55	4.39	4.68	0.37	0.96	0.59	0.87
Cooking loss (%)								
1-day postmortem	21.94	19.49	20.68	20.75	0.54	<0.01	0.94	0.16
5-day postmortem	21.09	20.38	20.89	20.58	0.62	0.42	0.73	0.56
Shear force (Kg)								
1-day postmortem	6.39	6.30	6.29	6.40	0.30	0.83	0.81	0.09
5-day postmortem	5.77	5.49	5.56	5.70	0.29	0.50	0.72	0.06
Sarcomere length (μm)	2.50	2.53	2.50	2.54	0.05	0.68	0.61	0.89
Muscle fiber diameter (μm)	80.75	74.71	76.38	79.08	4.90	0.39	0.70	0.81

Note: MLAB, multi-lactic acid bacterial; pH_45_, pH at 45 min postmortem; pH_u_, ultimate pH; L*, lightness; a*, redness; b*, yellowness; WHC, water holding capacity. A *p*-Value < 0.05 was considered statistically significant.

**Table 5:** Effects of multi-lactic acid bacterial (MLAB) probiotic supplementation on nutritional composition and ribonucleotide content of *Longissimus thoracis* muscle in fattening pigs.

Items	Treatment (T)		Sex (S)		SE	*p*-Value		
	Control	MLAB	Barrow	Female		T	S	T × S
Nutrient composition (%)								
Moisture	71.64	71.58	71.26	71.96	0.38	0.92	0.21	0.36
Ash	1.20	1.21	1.20	1.23	0.01	0.65	0.07	0.08
Fat	3.49	3.56	3.88	3.17	0.26	0.85	0.06	0.24
Protein	24.01	22.89	22.80	24.10	0.40	0.06	0.03	0.92
Ribonucleotides (mg/100 g)								
Hypoxanthine	24.76	26.05	25.57	25.24	1.40	0.52	0.89	0.41
Inosine	101.01	105.61	106.66	99.96	6.74	0.64	0.49	0.94
Inosine 5ʹ-monophosphate	209.63	230.55	207.97	232.21	13.53	0.32	0.26	0.22
Guanosine 5ʹ-monophosphate	9.21	10.09	11.75	7.55	1.75	0.73	0.11	0.53

Note: MLAB, multi-lactic acid bacterial; SE, standard error. A *p*-Value < 0.05 was considered statistically significant.

**Table 6:** Effects of multi-lactic acid bacterial (MLAB) probiotic supplementation on fatty acids of *Longissimus thoracis* muscle in fattening pigs.

Fatty Acid Composition (% of Fatty Acids)	Treatment (T)		Sex (S)		SE	*p*-Value		
	Control	MLAB	Barrow	Female		T	S	T × S
C14:0	1.33	1.32	1.36	1.28	0.05	0.82	0.34	0.47
C14:1	0.09	0.08	0.09	0.08	0.02	0.73	0.86	0.47
C15:0	0.03	0.03	0.03	0.03	0.01	0.95	0.55	0.10
C16:0	26.69	26.42	26.78	26.34	0.33	0.58	0.38	0.27
C16:1	3.20	3.39	3.36	3.23	0.13	0.32	0.50	0.85
C17:0	0.22	0.21	0.21	0.21	0.01	0.54	1.00	0.36
C17:1	0.26	0.24	0.24	0.25	0.02	0.54	0.94	0.75
C18:0	11.82	11.52	11.81	11.53	0.29	0.48	0.52	0.25
C18:1n9c	46.17	47.36	46.37	47.16	0.46	0.10	0.24	0.27
C18:2n6c	7.38	7.10	7.20	7.26	0.36	0.59	0.91	0.52
C20:0	0.20	0.19	0.19	0.20	0.01	0.53	0.80	0.60
C18:3n6	0.02	0.02	0.02	0.02	0.00	0.60	0.94	0.41
C20:1n9	0.87	0.76	0.79	0.84	0.06	0.22	0.60	0.30
C18:3n3	0.39	0.25	0.36	0.28	0.07	0.18	0.46	0.41
C21:0	0.02	0.02	0.02	0.02	0.00	0.37	0.40	0.70
C20:2	0.40	0.27	0.34	0.33	0.04	0.03	0.92	0.72
C22:0	0.02	0.03	0.03	0.02	0.01	0.80	0.73	0.79
C20:3n6	0.11	0.10	0.10	0.12	0.01	0.64	0.24	0.78
C22:1n9	0.01	0.01	0.11	0.01	0.00	0.51	0.84	0.26
C20:3n3	0.05	0.03	0.05	0.03	0.01	0.17	0.30	0.14
C20:4n6	0.63	0.62	0.59	0.67	0.08	0.93	0.45	0.74
C22:6n3	0.07	0.06	0.05	0.08	0.01	0.65	0.19	0.75
Saturated fatty acid; SFA	40.33	39.72	40.42	39.63	0.53	0.43	0.31	0.15
Monounsaturated fatty acid; MUFA	50.61	51.84	50.87	51.57	0.50	0.10	0.33	0.35
Polyunsaturated fatty acid; PUFA	9.05	8.44	8.70	8.79	0.43	0.34	0.89	0.46
PUFA:SFA	0.22	0.22	0.22	0.22	0.01	0.62	0.71	0.32

Note: MLAB, multi-lactic acid bacterial; SE, standard error. A *p*-Value < 0.05 was considered statistically significant.

**Table 7:** Effects of multi-lactic acid bacterial (MLAB) probiotic supplementation on total amino acids of *Longissimus thoracis* muscle in fattening pigs.

Total Amino Acids (mg/g)	Treatment (T)		Sex (S)		SE	*p*-Value		
	Control	MLAB	Barrow	Female		T	S	T × S
Essential amino acids								
Histidine	0.76	0.72	0.75	0.73	0.08	0.75	0.88	0.68
Arginine	0.26	0.27	0.28	0.25	0.03	0.80	0.47	0.93
Threonine	0.15	0.15	0.15	0.14	0.02	1.00	0.66	0.72
Lysine	0.19	0.17	0.19	0.18	0.03	0.46	0.76	0.22
Valine	0.11	0.14	0.14	0.11	0.02	0.34	0.38	0.78
Methionine	0.23	0.20	0.21	0.23	0.02	0.40	0.48	0.57
Leucine	0.55	0.58	0.60	0.53	0.08	0.78	0.55	0.72
Phenylalanine	2.12	2.03	2.15	2.01	0.28	0.82	0.72	0.83
Total essential amino acids	4.36	4.26	4.46	4.17	0.53	0.89	0.70	0.75
Non-essential amino acids								
Aspartic acid	0.16	0.16	0.17	0.15	0.02	1.00	0.49	0.95
Glutamic acid	4.34	2.77	4.47	2.64	1.20	0.37	0.29	0.33
Serine	0.14	0.14	0.15	0.14	0.01	1.00	0.44	0.28
Glycine	0.20	0.21	0.21	0.19	0.03	0.87	0.59	0.78
Alanine	0.36	0.36	0.38	0.34	0.06	0.92	0.64	0.49
Proline	0.30	0.30	0.30	0.29	0.01	0.82	0.60	0.82
Total non-essential amino acids	0.97	0.96	0.97	0.96	0.10	0.95	0.99	0.22

Note: MLAB, multi-lactic acid bacterial; SE, standard error. A *p*-Value < 0.05 was considered statistically significant.

**Table 8:** Effects of multi-lactic acid bacterial (MLAB) probiotic supplementation on free amino acids of *Longissimus thoracis* muscle in fattening pigs.

Free Amino Acids (mg/g)	Treatment (T)		Sex (S)		SE	*p*-Value		
	Control	MLAB	Barrow	Female		T	S	T × S
Essential amino acids								
Histidine	0.49	0.53	0.51	0.50	0.06	0.69	0.89	0.41
Threonine	0.06	0.07	0.08	0.05	0.01	0.45	0.05	0.48
Lysine	0.28	0.30	0.35	0.23	0.08	0.87	0.28	0.71
Valine	0.07	0.07	0.07	0.07	0.01	0.82	0.63	0.61
Methionine	0.05	0.05	0.05	0.05	0.01	1.00	0.81	0.47
Isoleucine	0.16	0.14	0.20	0.11	0.05	0.84	0.22	0.68
Phenylalanine	0.11	0.11	0.10	0.12	0.01	0.75	0.31	0.21
Leucine	0.19	0.26	0.25	0.19	0.06	0.38	0.46	0.81
Arginine	1.98	1.92	2.04	1.86	0.18	0.80	0.50	0.40
Total essential amino acids	3.39	3.44	3.66	3.18	0.34	0.91	0.34	0.55
Non-essential amino acids								
Aspartic acid	0.003	0.004	0.004	0.004	0.00	0.37	0.46	0.05
Glutamic acid	0.17	0.12	0.10	0.20	0.03	0.14	0.02	0.12
Serine	0.05	0.03	0.03	0.05	0.01	0.13	0.03	0.04
Glycine	0.14	0.13	0.13	0.14	0.02	0.67	0.74	0.38
Alanine	0.52	0.59	0.59	0.52	0.06	0.37	0.36	0.44
Proline	0.05	0.07	0.08	0.05	0.01	0.09	0.01	0.44
Tryptophan	0.04	0.02	0.04	0.02	0.02	0.45	0.42	0.32
Total non-essential amino acids	0.97	0.96	0.97	0.96	0.10	0.95	1.00	0.22
Human taste classification								
Umami	0.17	0.12	0.10	0.20	0.03	0.13	0.02	0.12
Sweetness	0.81	0.89	0.90	0.80	0.08	0.50	0.38	0.47
Bitterness	1.15	1.18	1.31	1.02	0.14	0.88	0.18	0.66

Note: MLAB, multi-lactic acid bacterial. Umami, aspartic acid+ glutamic acid. Sweetness, threonine+ serine+ glycine+ alanine+ proline. Bitterness, histidine+ threonine+ lysine+ valine+ methionine+ isoleucine+ tryptophan. SE, standard error. A *p*-Value < 0.05 was considered statistically significant.

**Table 9:** Effects of multi-lactic acid bacterial (MLAB) probiotic supplementation on intestinal morphology in fattening pigs.

Intestinal Morphology (µm)	Treatment (T)		Sex (S)		SE	*p*-Value		
	Control	MLAB	Barrow	Female		T	S	T × S
Duodenum								
VH	325.06	328.58	316.99	336.65	14.93	0.87	0.36	0.90
CD	207.19	216.98	187.12	237.05	17.42	0.69	0.05	0.29
VH:CD	1.67	1.72	1.91	1.48	0.09	0.77	0.03	0.20
Jejunum								
VH	331.68	369.18	354.20	346.66	15.49	0.10	0.73	0.84
CD	171.40	182.55	160.39	193.56	11.71	0.51	0.06	0.16
VH:CD	2.03	2.14	2.31	1.86	0.12	0.50	0.01	0.14
Ileum								
VH	342.91	330.21	325.14	347.98	10.04	0.38	0.12	0.70
CD	176.69	187.57	162.02	202.24	10.62	0.48	0.01	0.07
VH:CD	2.03	1.85	2.08	1.80	0.10	0.20	0.05	0.05

Note: MLAB, multi-lactic acid bacterial; VH, villus height; CD, crypt depth; SE, standard error. A *p*-Value < 0.05 was considered statistically significant.

## Data Availability

The original contributions presented in this study are included in the article/Supplementary Materials. Further inquiries can be directed to the corresponding author.

## Supplementary Materials

The following supporting information can be downloaded at: https://www.mdpi.com/article/10.3390/vetsci13040322/s1, Table S1: Top 10 of relative abundance (%) between two groups; Table S2: Significant differences in relative abundance (%) of microbial taxa between dietary groups at the family and genus levels.

## References

[1] Pereira W.A., Franco S.M., Reis I.L., Mendonca C.M.N., Piazentin A.C.M., Azevedo P.O.S., Tse M.L.P., De Martinis E.C.P., Gierus M., Oliveira R.P.S. (2022). Beneficial effects of probiotics on the pig production cycle: An overview of clinical impacts and performance. Vet. Microbiol..

[2] Ali M.S., Lee E.B., Hsu W.H., Suk K., Sayem S.A.J., Ullah H.M.A., Lee S.J., Park S.C. (2023). Probiotics and postbiotics as an alternative to antibiotics: An emphasis on pigs. Pathogens.

[3] Liu Y., Espinosa C.D., Abelilla J.J., Casas G.A., Lagos L.V., Lee S.A., Kwon W.B., Mathai J.K., Navarro D., Jaworski N.W. (2018). Non-antibiotic feed additives in diets for pigs: A review. Anim. Nutr..

[4] Kwak M.-J., Tan P.L., Oh J.K., Chae K.S., Kim J., Kim S.H., Eun J.-S., Chee S.W., Kang D.-K., Kim S.H. (2021). The effects of multispecies probiotic formulations on growth performance, hepatic metabolism, intestinal integrity and fecal microbiota in growing-finishing pigs. Anim. Feed. Sci. Technol..

[5] Liao S.F., Nyachoti M. (2017). Using probiotics to improve swine gut health and nutrient utilization. Anim. Nutr..

[6] Anee I.J., Alam S., Begum R.A., Shahjahan R.M., Khandaker A.M. (2021). The role of probiotics on animal health and nutrition. J. Basic. Appl. Zool..

[7] Shehata A.A., Yalcin S., Latorre J.D., Basiouni S., Attia Y.A., Abd El-Wahab A., Visscher C., El-Seedi H.R., Huber C., Hafez H.M. (2022). Probiotics, prebiotics, and phytogenic substances for optimizing gut health in poultry. Microorganisms.

[8] Sahatsanon K., Sivapirunthep P., Sringarm K., Arjin C., Hnokaew P., Chaweewan K., Chaosap C. (2025). Influence of host-specific and locally isolated multi-strain probiotics on piglet performance, mortality, inflammatory response, and gut microbiome. Anim. Biosci..

[9] Duan H., Lu L., Zhang L., Li J., Gu X., Li J. (2023). Effects of Lactobacillus Lactis supplementation on growth performance, hematological parameters, meat quality and intestinal flora in growing-finishing pigs. Animals.

[10] Tian Z., Cui Y., Lu H., Wang G., Ma X. (2021). Effect of long-term dietary probiotic Lactobacillus reuteri 1 or antibiotics on meat quality, muscular amino acids and fatty acids in pigs. Meat Sci..

[11] Zhu X., Liu B., Xiao J., Guo M., Zhao S., Hu M., Cui Y., Li D., Wang C., Ma S. (2022). Effects of different roughage diets on fattening performance, meat quality, fatty acid composition, and rumen microbe in steers. Front. Nutr..

[12] Kwoji I.D., Aiyegoro O.A., Okpeku M., Adeleke M.A. (2021). Multi-Strain probiotics: Synergy among isolates enhances biological activities. Biology.

[13] Faucitano L. (2018). Preslaughter handling practices and their effects on animal welfare and pork quality. J. Anim. Sci..

[14] Idrees M., Imran M., Atiq N., Zahra R., Abid R., Alreshidi M., Roberts T., Abdelgadir A., Tipu M.K., Farid A. (2022). Probiotics, their action modality and the use of multi-omics in metamorphosis of commensal microbiota into target-based probiotics. Front. Nutr..

[15] Lähteinen T., Rinttilä T., Koort J.M.K., Kant R., Levonen K., Jakava-Viljanen M., Björkroth J., Palva A. (2015). Effect of a multispecies lactobacillus formulation as a feeding supplement on the performance and immune function of piglets. Livest. Sci..

[16] Lefter N.A., Hăbeanu M., Gheorghe A., Dumitru M., Gal C., Vlaicu P.A. (2023). Effects of microencapsulated probiotics on performance, organ development, diarrhoea incidences, blood parameters, intestinal histomorphology and microflora in weaning piglets. Agriculture.

[17] Zhu Q., Song M., Azad M.A.K., Ma C., Yin Y., Kong X. (2022). Probiotics and synbiotics addition to Bama mini-pigs’ diet improve carcass traits and meat quality by altering plasma metabolites and related gene expression of offspring. Front. Vet. Sci..

[18] Saman P., Chaiongkarn A., Moonmangmee S., Singto S., Kornngam P., Yongkit N. (2022). Research and Development of Effective Mixed Microorganism for Piglet Production.

[19] Joysowal M., Saikia B.N., Dowarah R., Tamuly S., Kalita D., Choudhury K.B.D. (2018). Effect of probiotic Pediococcus acidilactici FT28 on growth performance, nutrient digestibility, health status, meat quality, and intestinal morphology in growing pigs. Vet. World.

[20] Li R., Liu J., Liu Y., Cao L., Qiu W., Qin M., Aluko R. (2023). Probiotic effects of Bacillus subtilis on growth performance and intestinal microecological balance of growing-to-finishing pigs. J. Food Biochem..

[21] Sanchai J. (2004). Meat Management.

[22] Chaosap C., Sivapirunthep P., Takeungwongtrakul S., Zulkifli R.B.M., Sazili A.Q. (2020). Effects of Zn-L-Selenomethionine on carcass composition, meat characteristics, fatty acid composition, glutathione peroxidase activity, and ribonucleotide content in broiler chickens. Food Sci. Anim. Resour..

[23] Lertpatarakomol R., Chaosap C., Chaweewan K., Sitthigripong R., Limsupavanich R. (2018). Carcass characteristics and meat quality of purebred Pakchong 5 and crossbred pigs sired by Pakchong 5 or Duroc boar. Asian Australas. J. Anim. Sci..

[24] Reuter G. Standardization of the filter press method with the “Braun schewiger apparalus and application of a new template evaluation system for rapid determination of unbond water in meat. Proceedings of the Meeting Biophysical PSE-Muscle analysis.

[25] AOAC (2006). Official Methods of Analysis.

[26] Chaosap C., Sahatsanon K., Sitthigripong R., Sawanon S., Setakul J. (2021). The effects of using pineapple stem starch as an alternative starch source and ageing period on meat quality, texture profile, ribonucleotide content, and fatty acid composition of Longissimus Thoracis of fattening dairy steers. Foods.

[27] (2005). First Edition Animal Feeding Stuffs-Determination of Amino Acids Content.

[28] Barfod K.K., Roggenbuck M., Hansen L.H., Schjørring S., Larsen S.T., Sørensen S.J., Krogfelt K.A. (2013). The murine lung microbiome in relation to the intestinal and vaginal bacterial communities. BMC Microbiol..

[29] Callahan B.J., McMurdie P.J., Rosen M.J., Han A.W., Johnson A.J., Holmes S.P. (2016). DADA2: High-resolution sample inference from Illumina amplicon data. Nat. Methods.

[30] Piao J.R., Tian J.Z., Kim B.G., Choi Y.I., Kim Y.Y., Han I.K. (2004). Effects of sex and market weight on performance, carcass characteristics and pork quality of market hogs. Asian-Australas. J. Anim. Sci..

[31] Schumacher M., DelCurto-Wyffels H., Thomson J., Boles J. (2022). Fat deposition and fat effects on meat quality-A review. Animals.

[32] Fusco W., Lorenzo M.B., Cintoni M., Porcari S., Rinninella E., Kaitsas F., Lener E., Mele M.C., Gasbarrini A., Collado M.C. (2023). Short-chain fatty-acid-producing bacteria: Key components of the human gut microbiota. Nutrients.

[33] Wang W., Dang G., Hao W., Li A., Zhang H., Guan S., Ma T. (2024). Dietary supplementation of compound probiotics improves intestinal health by modulated microbiota and its scfa products as alternatives to in-feed antibiotics. Probiot Antimicrob. Proteins.

[34] Sarmiento-Garcia A., Vieira-Aller C. (2023). Improving fatty acid profile in native breed pigs using dietary strategies: A review. Animals.

[35] Watanabe G., Motoyama M., Nakajima I., Sasaki K. (2018). Relationship between water-holding capacity and intramuscular fat content in Japanese commercial pork loin. Asian-Australas. J. Anim. Sci..

[36] Modzelewska-Kapituła M., Żmijewski T. (2023). Changes in water holding capacity and shear force in fallow deer muscles during ageing. Appl. Sci..

[37] Xia J.Q., Liu D.Y., Liu J., Jiang X.P., Wang L., Yang S., Liu D. (2023). Sex effects on carcass characteristics, meat quality traits and meat amino acid and fatty acid compositions in a novel Duroc line pig. J. Anim. Physiol. Anim. Nutr..

[38] Zhdanov D.V., Mykhalko O.H., Povod M.H., Zamaratskaia G. (2025). Carcass traits and meat quality of surgically castrated and immunocastrated pigs at two slaughter weights. Animals.

[39] Zheng A., Luo J., Meng K., Li J., Zhang S., Li K., Liu G., Cai H., LBryden W., Yao B. (2015). Proteome changes underpin improved meat quality and yield of chickens (Gallus gallus) fed the probiotic Enterococcus faecium. BMC Genom..

[40] Hernandez R.O., Rocha A.O., Cai C., Erasmus M., Johnson J.S., Brito L.F. (2025). Effects of microclimate during transport on physiological indicators of market pig welfare: A systematic review with meta-analysis. Front. Vet. Sci..

[41] Atuahene D., Sam B.A., Idan F., Sana S.S., Knop R., Suthar T., Kumar H., Shaikh A.M. (2025). Probiotics, prebiotics, and synbiotics in pigs and poultry: A review of gut health, performance, and environmental outcomes. Vet. Sci..

[42] Shaw F.D., Trout G.R. (1995). Plasma and muscle cortisol measm’ements as indicators of meat quaaty and stress in pigs. Meat Sci..

[43] Koomkrong N., Boonkaewwan C., Laenoi W., Kayan A. (2017). Blood haematology, muscle pH and serum cortisol changes in pigs with different levels of drip loss. Asian-Australas. J. Anim. Sci..

[44] Sardi L., Gastaldo A., Borciani M., Bertolini A., Musi V., Martelli G., Cavallini D., Rubini G., Nannoni E. (2020). Identification of possible pre-slaughter indicators to predict stress and meat quality: A study on heavy pigs. Animals.

[45] Sommavilla R., Faucitano L., Gonyou H., Seddon Y., Bergeron R., Widowski T., Crowe T., Connor L., Scheeren M.B., Goumon S. (2017). Season, transport duration and trailer compartment effects on blood stress indicators in pigs: Relationship to environmental, behavioral and other physiological factors, and pork quality traits. Animals.

[46] Ertbjerg P., Puolanne E. (2017). Muscle structure, sarcomere length and influences on meat quality: A review. Meat Sci..

[47] Chang S.Y., Belal S.A., Kang D.R., Il Choi Y., Kim Y.H., Choe H.S., Heo J.Y., Shim K.S. (2018). Influence of probiotics-friendly pig production on meat quality and physicochemical characteristics. Korean J. Food Sci. Anim. Resour..

[48] Lorenzo J.M., Sarries M.V., Franco D. (2013). Sex effect on meat quality and carcass traits of foals slaughtered at 15 months of age. Animal.

[49] Zmijewski T., Modzelewska-Kapitula M. (2021). The influence of age and sex on carcass characteristics and chemical composition of the longissimus thoracis et lumborum muscle in wild boars (*Sus scrofa*). Arch. Anim. Breed..

[50] Alves L.K.S., Carnino B.B., Muro B.B.D., Pairis-Garcia M.D., Dipold C.C., Dos Santos F.M., Lo Buono J.E.M., Garbossa P.L.M., Silva Junior F.V., Garbossa C.A.P. (2025). Performance, carcass, and pork traits in barrows and gilts slaughtered over 130 kg: Insights from a Brazilian perspective. Transl. Anim. Sci..

[51] Chaosap C., Buajoom W., Pothising N., Kongtasorn C., Adeyemi K.D. (2025). Effects of genotype and sex on carcass traits, myosin heavy chain isoforms and meat characteristics of pigs. Animals.

[52] Muroya S., Oe M., Ojima K., Watanabe A. (2019). Metabolomic approach to key metabolites characterizing postmortem aged loin muscle of Japanese black (Wagyu) cattle. Asian-Australas. J. Anim. Sci..

[53] Tikk M., Tikk K., Tørngren M., Meinert L., Aaslyng M., Karlsson A., Andersen H. (2006). Development of inosine monophosphate and its degradation products during aging of pork of different qualities in relation to basic taste and retronasal flavor perception of the meat. J. Agric. Food Chem..

[54] Dinh T.T.N., To K.V., Schilling M.W. (2021). Fatty acid composition of meat animals as flavor precursors. Meat Muscle Biol..

[55] Huang Y.S., Huang W.C., Li C.W., Chuang L.T. (2011). Eicosadienoic acid differentially modulates production of pro-inflammatory modulators in murine macrophages. Mol. Cell. Biochem..

[56] Wu T., Wang G., Xiong Z., Xia Y., Song X., Zhang H., Wu Y., Ai L. (2022). Probiotics interact with lipids metabolism and affect gut health. Front. Nutr..

[57] Grela E.R., Swiatkiewicz M., Florek M., Bakowski M., Skiba G. (2021). Effect of inulin source and a probiotic supplement in pig diets on carcass traits, meat quality and fatty acid composition in finishing pigs. Animals.

[58] Kutay H., Şahan Z., Polat Açık İ., Durmuş M. (2024). Factors affecting meat quality in farm animals. BIO Web Conf..

[59] Ma X., Yu M., Liu Z., Deng D., Cui Y., Tian Z., Wang G. (2020). Effect of amino acids and their derivatives on meat quality of finishing pigs. J. Food Sci. Technol..

[60] Yang M., Vousden K.H. (2016). Serine and one-carbon metabolism in cancer. Nat. Rev. Cancer.

[61] Qin C., Huang P., Qiu K., Sun W., Xu L., Zhang X., Yin J. (2015). Influences of dietary protein sources and crude protein levels on intracellular free amino acid profile in the longissimus dorsi muscle of finishing gilts. J. Anim. Sci. Biotechnol..

[62] Chen X., Cao J., Chang C., Geng A., Wang H., Chu Q., Yan Z., Zhang X., Zhang Y., Liu H. (2023). Effects of age on compounds, metabolites and meat quality in Beijing-You chicken breast meat. Animals.

[63] Shin D., Chang S.Y., Bogere P., Won K., Choi J.Y., Choi Y.J., Lee H.K., Hur J., Park B.Y., Kim Y. (2019). Beneficial roles of probiotics on the modulation of gut microbiota and immune response in pigs. PLoS ONE.

[64] Wang X., Yin L., Geng C., Zhang J., Li J., Huang P., Li Y., Wang Q., Yang H. (2025). Impact of different feed intake levels on intestinal morphology and epithelial cell differentiation in piglets. J. Anim. Sci..

[65] Palma-Granados P., Lara L., Seiquer I., Lachica M., Fernandez-Figares I., Haro A., Nieto R. (2021). Protein retention, growth performance and carcass traits of individually housed immunocastrated male- and female- and surgically castrated male Iberian pigs fed diets of increasing amino acid concentration. Animal.

[66] Marchewka J., Sztandarski P., Zdanowska-Sasiadek Z., Adamek-Urbanska D., Damaziak K., Wojciechowski F., Riber A.B., Gunnarsson S. (2021). Gastrointestinal tract morphometrics and content of commercial and indigenous chicken breeds with differing ranging profiles. Animals.

[67] Cavallini D., Lamanna M., Colleluori R., Silvestrelli S., Ghiaccio F., Buonaiuto G., Formigoni A. (2025). The use of rumen-protected amino acids and fibrous by-products can increase the sustainability of milk production. Front. Vet. Sci..

[68] Ma T., Huang W., Li Y., Jin H., Kwok L.Y., Sun Z., Zhang H. (2023). Probiotics alleviate constipation and inflammation in late gestating and lactating sows. NPJ Biofilms Microbiomes.

[69] NCBI:txid2005523 (2022). Taxanomy Brower: Paludibacteraceae. https://www.ncbi.nlm.nih.gov/Taxonomy/Browser/wwwtax.cgi?mode=Info&id=2005523&lvl=3&p=has_linkout&p=blast_url&p=genome_blast&lin=f&keep=1&srchmode=1&unlock.

[70] Azziz G., Gimenez M., Carballo C., Espino N., Barlocco N., Batista S. (2023). Characterization of the fecal microbiota of Pampa Rocha pigs, a genetic resource endemic to eastern Uruguay. Heliyon.

[71] Ueki A., Akasaka H., Suzuki D., Ueki K. (2006). Paludibacter propionicigenes gen. nov., sp. nov., a novel strictly anaerobic, gram-negative, propionate-producing bacterium isolated from plant residue in irrigated rice-field soil in Japan. Int. J. Syst. Evol. Microbiol..

[72] Ma J., Piao X., Mahfuz S., Long S., Wang J. (2022). The interaction among gut microbes, the intestinal barrier and short chain fatty acids. Anim. Nutr..

[73] BiologyInsights (2024). Oxalobacter Formigenes: Key Player in Gut Health and Oxalate Balance. https://biologyinsights.com/oxalobacter-formigenes-key-player-in-gut-health-and-oxalate-balance/.

[74] Rahman M.M., Abdullah R.B., Wan Khadijah W.E. (2013). A review of oxalate poisoning in domestic animals: Tolerance and performance aspects. J. Anim. Physiol. Anim. Nutr..

