# Supplementary material for: Effects of Host-Specific Multi-Lactic Acid Bacterial Probiotics on Performance, Carcass Traits, Meat Quality, and Gut Microbiome in Fattening Pigs
Source: Vet Sci. 2026 Mar 26;13(4):322. doi: 10.3390/vetsci13040322 (PMC13119831; doi:10.3390/vetsci13040322)
Supplement: Supplementary file 1 [file vetsci-13-00322-s001.zip › Supplementary Table - MLAB -vetsci.pdf]

Supplementary Table S1. Top 10 of relative abundance (%) between two groups.

Taxon	Control	MLAB
Phylum		
Firmicutes	61.36	58.28
Bacteroidota	28.13	31.33
Proteobacteria	3.84	4.38
Euryarchaeota	3.10	2.23
Spirochaetota	1.89	1.63
Desulfobacterota	0.90	1.00
Campylobacterota	0.16	0.27
Verrucomicrobiota	0.20	0.21
Cyanobacteria	0.11	0.19
Actinobacteriota	0.15	0.11
Genus		
Muribaculaceae	9.34	9.77
Lactobacillus	13.24	5.77
Prevotella	8.01	8.11
Oscillospira	4.37	6.21
Succinivibrio	3.56	4.03
Prevotellaceae_NK3B31_group	3.27	3.46
Streptococcus	3.47	2.95
UCG-002	2.61	3.05
Methanobrevibacter	3.10	2.23
UCG-005	2.82	2.40

Note: MLAB, multi-lactic acid bacterial probiotics were added to pig diets.

Supplementary Table S2. Top 5 genera with significant differences between two groups (Mann–Whitney U Test).

Taxon	Control	MLAB	SEM	*p*-Value
Candidatus_Soleaferrea	0.080^{b}	0.191^{a}	0.017	0.001
Oxalobacter	0.005^{b}	0.028^{a}	0.005	0.013
Lachnoclostridium	0.039^{b}	0.076^{a}	0.008	0.028
Intestinimonas	0.002^{b}	0.009^{a}	0.002	0.037
*Clostridium_sensu_stricto*_6	0.120^{a}	0.038^{b}	0.023	0.049

Note: MLAB, multi-lactic acid bacterial probiotics were added to pig diets; SEM, standard error of the mean.

^{a,b} Different superscripts within the same row denote significant differences ($p < 0.05$).
